# Multi-omics approaches for drug-response characterization in primary biliary cholangitis and autoimmune hepatitis variant syndrome

**DOI:** 10.1186/s12967-024-05029-6

**Published:** 2024-02-29

**Authors:** Fan Yang, Leyu Zhou, Yi Shen, Xianglin Wang, Xiaoli Fan, Li Yang

**Affiliations:** Department of Gastroenterology and Hepatology and Sichuan University-University of Oxford Huaxi Joint Centre for Gastrointestinal Cancer, West China Hospital, Sichuan University, #37 Guoxue Road, Chengdu, 610041 Sichuan China

**Keywords:** Autoimmune liver diseases, Drug-response, Whole-transcriptomic, Metabolomic, Cytokinomic

## Abstract

**Background:**

Primary biliary cholangitis (PBC) and autoimmune hepatitis (AIH) variant syndrome (VS) exhibit a complex overlap of AIH features with PBC, leading to poorer prognoses than those with PBC or AIH alone. The biomarkers associated with drug response and potential molecular mechanisms in this syndrome have not been fully elucidated.

**Methods:**

Whole-transcriptome sequencing was employed to discern differentially expressed (DE) RNAs within good responders (GR) and poor responders (PR) among patients with PBC/AIH VS. Subsequent gene ontology (GO) analysis and Kyoto Encyclopedia of Genes and Genomes (KEGG) pathway analysis were conducted for the identified DE RNAs. Plasma metabolomics was employed to delineate the metabolic profiles distinguishing PR and GR groups. The quantification of immune cell profiles and associated cytokines was achieved through flow cytometry and immunoassay technology. Uni- and multivariable logistic regression analyses were conducted to construct a predictive model for insufficient biochemical response. The performance of the model was assessed by computing the area under the receiver operating characteristic (AUC) curve, sensitivity, and specificity.

**Findings:**

The analysis identified 224 differentially expressed (DE) mRNAs, 189 DE long non-coding RNAs, 39 DE circular RNAs, and 63 DE microRNAs. Functional pathway analysis revealed enrichment in lipid metabolic pathways and immune response. Metabolomics disclosed dysregulated lipid metabolism and identified PC (18:2/18:2) and PC (16:0/20:3) as predictors. CD4^+^ T helper (Th) cells, including Th2 cells and regulatory T cells (Tregs), were upregulated in the GR group. Pro-inflammatory cytokines (IFN-γ, TNF-α, IL-9, and IL-17) were downregulated in the GR group, while anti-inflammatory cytokines (IL-10, IL-4, IL-5, and IL-22) were elevated. Regulatory networks were constructed, identifying CACNA1H and ACAA1 as target genes. A predictive model based on these indicators demonstrated an AUC of 0.986 in the primary cohort and an AUC of 0.940 in the validation cohort for predicting complete biochemical response.

**Conclusion:**

A combined model integrating genomic, metabolic, and cytokinomic features demonstrated high accuracy in predicting insufficient biochemical response in patients with PBC/AIH VS. Early recognition of individuals at elevated risk for insufficient response allows for the prompt initiation of additional treatments.

**Supplementary Information:**

The online version contains supplementary material available at 10.1186/s12967-024-05029-6.

## Introduction

Autoimmune hepatitis (AIH) is a severe and chronic progressive inflammatory liver ailment characterized by an enduring autoimmune response directed against liver autoantigens [1, 2]. Primary cholestatic hepatitis (PBC) manifests as a chronic, cholestatic autoimmune liver condition characterized by the destruction of biliary epithelial cells and the presence of antimitochondrial antibodies (AMA) [3, 4]. PBC/AIH variant syndrome (VS) represents a variant form of either AIH or PBC, encompassing AIH features with cholestatic attributes or elevated aminotransferases in the context of PBC, accompanied by autoantibodies and elevated Immunoglobulin G (IgG) levels [5–8]. The manifestation of variant syndromes occurs often sequentially over time with no overlapping features at the initial diagnosis. PBC/AIH VS remains an uncommon disorder, affecting about 5–20% of patients with diagnostic features of either AIH or PBC [9]. This wide range reflects the lack of standardization of criteria used to define PBC/AIH overlapping disease. From a biological perspective, it remains unclear whether these syndromes should be considered unique autoimmune entities causing damage to bile ducts and hepatocytes or whether they represent presentations of two distinct diseases (PBC and AIH).

Based on the histologic, biochemical, and immunologic features of each parent disorder, the Paris criteria represent the most widely accepted diagnostic method for PBC/AIH VS [10]. The majority of investigators have opted for a first-line treatment regimen that combines ursodeoxycholic acid (UDCA) and corticosteroids, with or without azathioprine (AZA) [10–17]. Nevertheless, patients with PBC/AIH VS often experience more unfavorable prognoses compared to those with PBC or AIH alone [18]. A retrospective cohort study in Korea demonstrated that overlap syndrome patients exhibited a lower remission rate to UDCA and steroid combination therapy, coupled with a significantly shorter time-to-progression of liver disease than AIH patients [19]. Our previous study, encompassing 432 PBC patients, revealed that the biochemical remission rate of PBC patients with AIH features was markedly lower than that of patients with PBC alone, with 88.9% of liver biopsy specimens from non-responders showing interface hepatitis [20]. In a retrospective study of a substantial PBC/AIH patient cohort, UDCA alone failed to induce a biochemical response in the majority of patients with interface hepatitis [13]. Despite this, there is a dearth of studies concerning the response in this patient subgroup. Our earlier investigation, which involved 134 PBC-AIH VS patients, indicated a significantly higher incidence of liver-related adverse events in non-responders compared to responders, with high cholesterol levels, reduced histologic bile ducts, and cirrhosis being potential risk factors for poor response [21]. The development of PBC/AIH VS is a multifaceted, multistep process involving various molecular pathways and factors [5]. Identifying more detailed regulatory molecules is imperative to better predict drug response in these patients, facilitating early intervention.

The advancement and decreasing costs of molecular measurement technologies have enabled the diverse profiling of disease molecular features, spanning the genome, transcriptome, proteome, metabolome, single-cell sequencing, or cytokinome [22, 23]. Whole-transcriptome analyses have revealed that a substantial portion of the human genome undergoes transcription, generating non-coding (nc) transcripts, such as long noncoding RNAs (lncRNAs), microRNAs (miRNAs), and circular RNAs (circRNAs) [24]. MiRNAs execute their biological roles by binding to miRNA response elements (MREs) on target mRNA, inducing gene silencing post-transcription [25]. LncRNAs have been implicated in modulating various cell phenotypes by influencing miRNA and mRNA expression and stability [26]. CircRNAs, owing to their resistance to exonucleases and enhanced stability compared to linear RNAs, can continuously accumulate within cells [27]. A comprehensive understanding of the molecular mechanisms and intricate network of RNA-level interactions could unveil innovative diagnostic and prognostic biomarkers [28, 29]. However, integrating this knowledge with other "omic" studies may provide a more profound understanding of disease processes [30, 31]. Metabolic profiling, or metabolomics, provides information-rich insights into metabolic alterations reflective of genetic, epigenetic, and environmental influences on cellular physiology [32, 33]. Cytokines, contributing to disease development and progression, exhibit deregulated serum levels in various liver diseases, correlating with patient outcomes [34, 35]. The integration of transcriptomics with metabolomics or cytokinomics holds the potential to offer deeper insights into disease pathogenesis compared to either approach in isolation [36].

In this study, we utilized whole-transcriptome sequencing to discern driver genes exhibiting notable transcriptional variations among PBC/AIH VS patients classified as good responders and poor responders. This thorough analysis sought to identify differentially expressed genes and non-coding RNAs (ncRNAs) linked to distinct metabolic and cytokine shifts in PBC/AIH VS. The discerned genetic, metabolic, and cytokine modifications were subsequently authenticated in an independent cohort. Additionally, a combined model integrating transcriptomic, metabolic, and cytokine data was formulated to predict an inadequate biochemical response to drug treatment in PBC/AIH VS patients.

## Methods

### Patient enrollments

This study enrolled 70 patients diagnosed with PBC/AIH VS at the West China Hospital of Sichuan University from October 2018 to June 2023. The diagnosis adhered to the Paris criteria [10] or, for PBC patients, was confirmed when distinctive AIH features were present. These features included sustained elevations in serum transaminase levels exceeding threefold the upper limit of normal (ULN) for at least 3 months, serum immunoglobulin G (IgG) levels surpassing 1.3-fold the ULN, and/or histological evidence demonstrating moderate to severe interface hepatitis. Exclusion criteria comprised (1) a variant syndrome with primary sclerosing cholangitis [37] or other chronic liver diseases; (2) acute liver failure; (3) severe dysfunction of other organs or systemic infection; (4) follow-up less than 6 months after starting immunosuppressants. The primary cohort consisted of 50 patients, and their clinical characteristics are detailed in Additional file 1: Table S1. Additional file 1: Fig. S1 presents a histopathological feature of PBC/AIH OS. Treatment strategies involved administering UDCA alongside methylprednisolone, either as a sole therapy or in combination with AZA or other immunosuppressive agents, following established guidelines [38]. A "good responder" (GR) achieved complete biochemical remission within 6 months of initiating immunosuppressive therapy, demonstrated by the restoration of normal serum aminotransferase and IgG levels. Patients not meeting these criteria were categorized as "poor responders" (PR). Additional file 1: Table S2 provides a comparative analysis of the baseline clinical characteristics of the two groups. Additionally, 20 patients constituted the independent validation cohort, and their clinical characteristics were not significantly different from the primary cohort, as shown in Additional file 1: Table S3. Ethical approval for this research was obtained from the Ethics Committee of the West China Hospital of Sichuan University, and informed consent was acquired from each participating patient.

### RNA sequencing

Total RNA was extracted from liver tissues of 5 patients each from the GR and PR groups using TRIzol reagent (Tiangen, China). Subsequently, RNA purity and concentration were evaluated using a NanoPhotometer® spectrophotometer. The integrity and quantity of RNA were assessed with the RNA Nano 6000 Assay Kit on the Bioanalyzer 2100 system. Fragment size selection was accomplished through agarose gel electrophoresis. Illumina HiSeqTM 4000 and Illumina HiSeqTM 2500 platforms were employed for whole transcriptome and small RNA sequencing, respectively. Whole transcriptome sequencing utilized paired-end sequencing (2 × 150 bp), while small RNA sequencing involved single-end sequencing (50 bp). The data obtained from whole transcriptome sequencing were employed to quantify the expression levels of mRNAs, lncRNAs, and circRNAs. Similarly, small RNA sequencing data were used to assess the expression levels of miRNAs.

### Differential expression analysis

The sequencing reads of mRNA, lncRNA, circRNA, and miRNA underwent alignment using the reference genome from the Genome Reference Consortium Human Build 38 Organism (GRCh38, GENCODE—https://www.gencodegenes.org/) [39]. Annotation files for mRNA and lncRNA were obtained from GENCODE, while miRNA annotation files were sourced from miRBase. Quantification of mRNA and circRNA expression levels employed StringTie, and FeatureCounts was used for miRNA and lncRNA quantification based on their respective transcript characteristics. DESeq2 was applied for conducting differential expression analysis on the quantified transcripts. Bowtie facilitated the alignment of sequencing data from miRNA libraries, while HISAT2 was employed for mRNA and lncRNA alignment. BWA was utilized for circRNA alignment. Specifically, CIRI2 and find_circ were used for circRNA quantification and expression analysis. Genes exhibiting a p-value < 0.05 and an absolute log2-fold change (|log2FC|) ≥ 1 were considered differentially expressed (DE) between the GR and PR groups in this study.

### Gene ontology and pathway analysis

The study employed Gene Ontology (GO, http://www.geneontology.org) analysis to evaluate the potential enrichment of differentially expressed genes in specific biological processes. The GO categories encompass three structured networks, delineated as Cellular Component (CC), Molecular Function (MF), and Biological Process (BP). Concurrently, pathway enrichment analysis utilized the Kyoto Encyclopedia of Genes and Genomes (KEGG, https://www.genome.jp/kegg). To mitigate the impact of multiple testing on enrichment results, the Benjamini & Hochberg procedure was implemented, and statistical significance was determined by an adjusted P-value < 0.05 [40].

### Competitive endogenous RNA (ceRNA) network construction

Our approach to constructing ceRNA networks involved integrating co-expression relationships between lncRNA-mRNA and circRNA-mRNA pairs, coupled with regulatory connections encompassing DE miRNAs and their target DE mRNAs and DE lncRNAs. The co-expression network was established by computing Pearson correlation coefficients and their corresponding p-values for multiple genes. Transcripts underwent stringent filtering criteria, preserving only those with a correlation coefficient (COR) > 0.85 and a p-value < 0.05. Our focus was on identifying positively correlated expression patterns among circRNA-mRNA and lncRNA-mRNA pairs. Additionally, we identified miRNAs capable of concurrently regulating both lncRNAs/circRNAs and mRNAs. Subsequently, our analysis extended to establishing positively correlated co-expression relationships between miRNA-regulated mRNAs and lncRNAs/circRNAs. Networks for lncRNA-miRNA-mRNA and circRNA-miRNA-mRNA were constructed using Cytoscape (http://www.cytoscape.org/).

### Quantitative real-time PCR analysis (RT-qPCR)

To validate the observed differential expression levels in liver tissues between the GR and PR groups, RT-qPCR was employed. Total RNA was extracted from liver tissues of 48 patients (27 GRs and 21 PRs). Subsequently, 1 μg of extracted total RNA underwent cDNA synthesis using the PrimeScript RT reagent kit (Takara, Japan). The resulting cDNA was subjected to real-time qPCR amplification utilizing SYBR Green Supermix on a CFX96 RT-qPCR detection system (BioRad, United States). The amplification protocol included an initial denaturation step at 95 °C for 3 min, followed by 39 cycles of 95 °C for 10 s, 60 °C for 20 s, and 72 °C for 20 s, concluding with 65 °C for 5 min in a 10 μl reaction volume [41]. To ensure quantification precision, the expression levels of genes were normalized to the expression level of actin. All primer used were listed in Additional file 1: Table S4 and were obtained from Tsingke (China).

### lc/ms-based metabolomics analysis

Serum samples underwent preparation through centrifugation at 2000*g* for 10 min. We collected plasma samples from 20 GRs and 30 PRs for metabolomic analysis. The resultant serum was transferred to Eppendorf tubes and reconstituted in pre-chilled 80% methanol by thorough vortexing. Following this, the samples were incubated on ice for 5 min, succeeded by high-speed centrifugation at 15,000×*g* at 4 °C for 20 min. The clarified samples were then transferred to fresh Eppendorf tubes and subjected to an additional centrifugation step at 15,000×*g* for 20 min before being readied for injection into the LC/MS system for analysis [42]. LC/MS analyses were performed using an ExionLCTM AD system (SCIEX) coupled with a QTRAP® 6500 + mass spectrometer (SCIEX), a process expertly executed at Novogene Co., Ltd. (Beijing, China). For the identification and annotation of detected metabolites, the KEGG, HMDB (http://www.hmdb.ca/), and Lipidmaps (http://www.lipidmaps.org/) databases were utilized. Both Principal Component Analysis (PCA) and Partial Least Squares Discriminant Analysis (PLS-DA) were conducted using metaX [43, 44]. Metabolites with a Variable Importance in Projection (VIP) score > 1.0, FC > 1.2 or FC < 0.833 and a p-value < 0.05 were considered as differential metabolites [45, 46]. Moreover, to elucidate the functional relevance of these metabolites and their associated metabolic pathways, in-depth investigations were carried out using the KEGG database.

### Cytokinome evaluation

Cytokine concentrations in plasma were assessed employing the LEGENDplex Human Th Cytokine Panel (BioLegend, United States) [47], adhering to the manufacturer's recommended protocols. In brief, plasma samples were incubated with beads coated with capture antibodies specific for IL-2, IL-4, IL-5, IL-6, IL-9, IL-10, IL-13, IL-17 A, IL-17 F, IL-22, IFN-γ, and TNF-α for 2 h at room temperature on a shaker. Following incubation, beads were washed and incubated with biotin-labeled detection antibodies for 1 h, followed by a final incubation with streptavidin-PE for 30 min at room temperature on a shaker. The beads were then washed and resuspended using washing buffer. Flow cytometry analysis was conducted using a FACS Canto cytometer, and data analysis was performed with the LEGENDplex analysis software, which differentiated between the 12 analytes based on bead size and internal dye. These cytokines are secreted by T helper (Th) cells. Correlation analyses were carried out using the R package corrplot to explore relationships and associations within the cytokine data.

### Flow cytometric analysis

Peripheral blood mononuclear cells were isolated from patients using human lymphocyte separation medium (Dakewei, China) and underwent a meticulous washing procedure with phosphate-buffered saline (PBS, Servicebio, China) [48]. Approximately 1 × 10^^6^ prepared cells were suspended in 100 μl of PBS and subsequently stained with a variety of fluorochrome-coupled antibodies. Staining was carried out for 30 min at 4 °C [49]. For intracellular cytokine and transcription factor staining [50], the incubated cells were collected, fixed for 10 min, and then permeabilized using intracellular fixation and permeabilization buffer (Thermo Fisher). Permeabilized cells were incubated with the respective monoclonal antibodies at 4 °C for 30 min. Following the staining procedure, cells were washed twice with PBS. Flow cytometric analysis was conducted using either an LSRFortessa flow cytometer (BD Bioscience, United States) or a CytoFLEX flow cytometer (Beckman, United States). Data analysis was executed using FlowJo software (TreeStar, United States). The antibodies utilized for flow cytometry analysis were all obtained from BioLegend (United States), including CD3-APC, CD4-BV650, CD25-PE, CD69-BV605, Foxp3-AF647, IL-17-BV421, IFN-γ-PerCP-Cy5.5, IL-4-PE-Cy7, live/dead-APC-Cy 7, CD56-PE, CD8-APC-Cy7, and DAPI-PB450, for the detection of T effector (Teff) cells, Th cells and natural killer (NK) cells.

### Multiplex immunofluorescence (MIF) staining

Liver tissues from 5 GRs and 5 PRs were collected for MIF staining [51]. The MIF staining procedure entailed the preparation of 4 µm sections from entire FFPE liver tissue blocks, followed by dewaxing and fixation using 10% neutralized formaldehyde. Antigen retrieval was achieved by subjecting the samples to heating in citrate buffer (pH 6.0) and/or Tris–EDTA buffer (pH 8.0) for 15 min. Each section underwent four consecutive rounds of antibody staining, with the initial staining conditions established for each specific primary antibody and subsequent optimization (Biossci, Wuhan, China). Quantitative assessment of MIF staining results was conducted based on the proportion of cell subsets, including CD4^+^, CD8^+^ and CD56^+^ cells, within the liver tissues under investigation.

### Statistical analysis

Comprehensive statistical analyses were undertaken utilizing GraphPad Prism 9, SPSS (version 25), and the R software (version 4.1.0). Continuous variables, reported as the mean ± standard, were compared using Student’s t test or the Mann–Whitney U test. For comparing categorical variables between the two groups, Pearson’s χ2 test and Fisher’s exact test were employed. Pearson's correlation test assessed correlations. Discriminative performance for each indicator was evaluated through the area under the receiver operating characteristic curve (AUC), sensitivities, and specificities. Univariable and multivariable logistic regression analyses identified clinical indicators, genes, metabolites, and cytokines for biochemical response and formed the predictive model. Variables with p < 0.05 in univariable analyses and AUC > 0.7 were included in multivariable analysis using a forward stepwise method. A nomogram was constructed for model visualization and utility based on regression coefficients. AUC assessed discriminative performance of a combined model using five indicators. Calibration curves were plotted for model calibration. The Hosmer–Lemeshow (H–L) test evaluated goodness of fit between observed and predicted values in models. Statistical significance was set at p < 0.05.

## Results

### Screening of differentially expressed RNAs in PR and GR groups

**Fig. 1:**
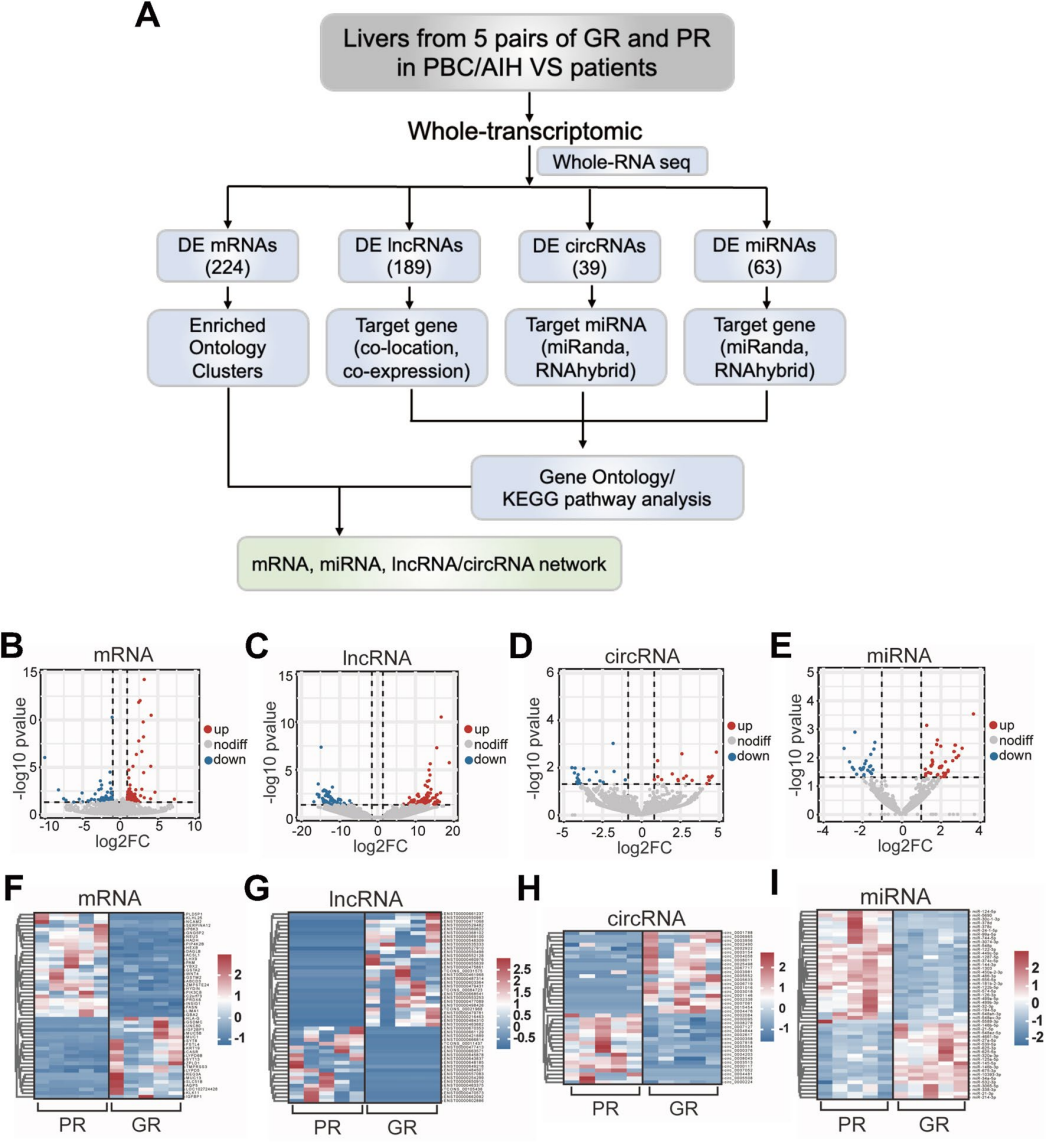
Screening of differentially expressed RNAs in GR and PR groups. **A** The flow chart of the whole-transcriptomic design. **B**–**E** The volcano plots of differential expressed mRNAs, lncRNAs, circRNAs and miRNAs. **F**–**I** The heatmaps of differential expressed mRNAs, lncRNAs, circRNAs and miRNAs

To investigate distinctive molecular profiles associated with treatment response in the liver tissues of patients with PBC-AIH VS, we conducted comprehensive whole-transcriptomic sequencing on liver tissues obtained from five pairs of GR and PR patients (Fig. 1A). Rigorous quality assessments, including Pearson correlation analysis and PCA analysis, were performed on these samples, and the results are presented in Additional file 1: Fig. S2A and S2B. RNAs meeting the criteria of a p-value < 0.05 and |log2FC|≥ 1 were considered differentially expressed between the GR and PR groups in this study. Our findings unveiled 224 DE mRNAs, 189 DE lncRNAs, 39 DE circRNAs, and 63 DE miRNAs, as illustrated in Fig. 1B–E. Heatmaps were generated to visually represent the expression patterns of clustered genes between the GR and PR groups, highlighting the top 50 DE mRNAs, lncRNAs, and miRNAs, along with all DE circRNAs (Fig. 1F–I). Subsequent analyses involved a detailed examination of significantly different genes through GO and KEGG pathway analyses. Moreover, regulatory networks were established, depicting interactions among mRNAs, miRNAs, and lncRNAs/circRNAs.

### Functional enrichment analysis of differentially expressed RNAs

**Fig. 2:**
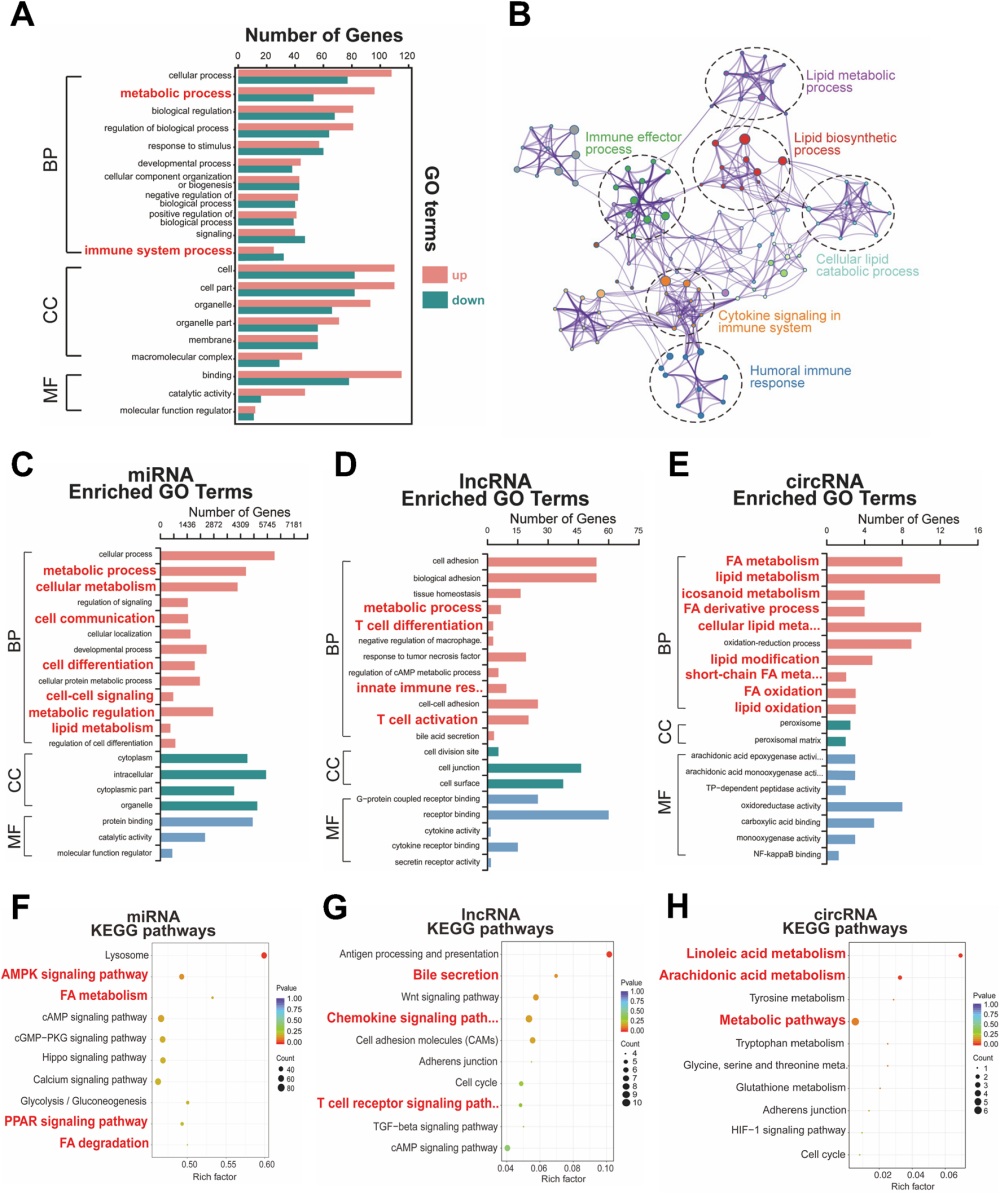
GO and KEGG pathway analysis of DE mRNAs, miRNAs, lncRNAs and circRNAs. **A** GO analysis of DE mRNAs. **B** Pathway analysis of DE mRNAs. **C**–**E** GO analysis of DE miRNAs, lncRNAs and circRNAs. **F–H** KEGG pathway analysis of DE miRNAs, lncRNAs and circRNAs

To elucidate the primary biological processes involving the identified differential genes, we conducted comprehensive GO and KEGG pathway analyses across multiple categories, providing detailed functional and characterization annotations. GO analysis revealed that BPs associated with DE mRNAs were significantly enriched in metabolic and immune system processes, visually depicted in Fig. 2A. Subsequent KEGG pathway analysis unveiled the enrichment of mRNA target genes in pathways primarily linked to hepatic metabolism and immune response. These pathways included lipid biosynthesis and metabolism, humoral immune responses, immune effector processes, and cytokine signaling, as illustrated in Fig. 2B. To explore the potential involvement of ncRNA-mediated regulatory networks in these pathways, we performed GO and KEGG analyses on DE miRNAs, lncRNAs, and circRNAs. The analyses associated with DE miRNAs demonstrated enrichment in metabolic processes, particularly fatty acid (FA) metabolism, and cell activities such as cell differentiation and various signaling pathways (Fig. 2C and F). DE lncRNAs were predominantly associated with immune response, encompassing T cell differentiation, activation, and T cell signaling pathways (Fig. 2D and G). Likewise, DE circRNAs exhibited enrichment in FA metabolism and related metabolic pathways (Fig. 2E and 2H). These results collectively indicate that the reprogramming of FA metabolism and T cell-mediated immune responses jointly contribute to the response process in VS patients. Previous studies have highlighted significant disorders in lipid metabolism in PBC patients, linked to disease progression [52], and emphasized the central role of T cells in the immune mechanism of AIH [53]. However, there is a paucity of research focusing on the metabolic and immunologic distinctions in different response settings among PBC/AIH VS patients. Therefore, our study provides a more in-depth exploration of both aspects.

### Dysregulated lipid metabolism is linked to the response of patients with VS

**Fig. 3:**
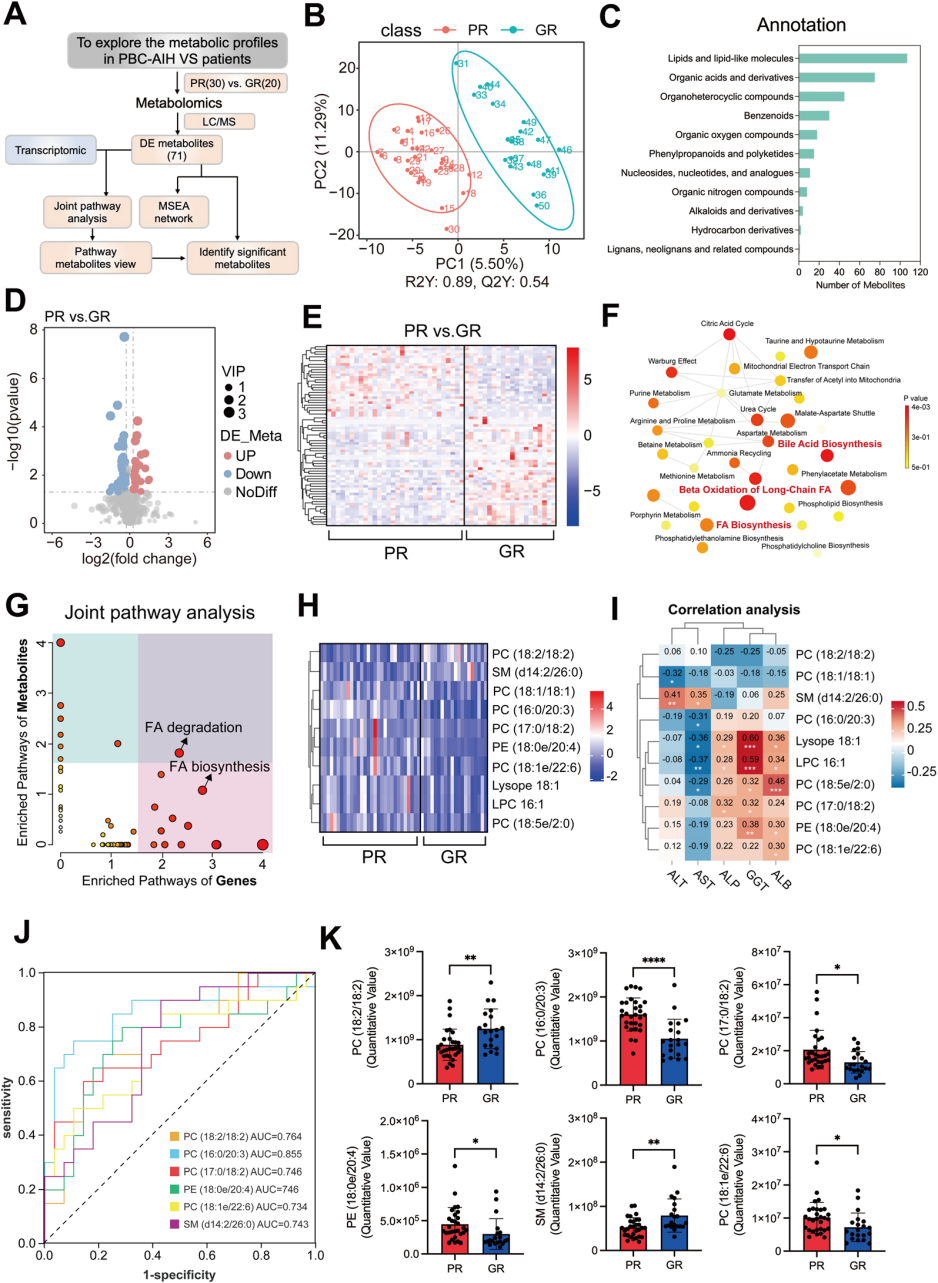
Metabolic profiles of PBC/AIH VS patients between PR and GR groups. **A** The flow chart of the metabolic design. **B** PCA analysis. **C** The annotation of the detected metabolites. **D**, **E** The volcano plot and heatmap of differential metabolites. **F** The enriched pathway analysis of the differential metabolites. **G** The joint pathway analysis of differential metabolites and genes. **H** Heatmap of the differential metabolites involved in the joint pathway analysis. **I** Correlation analysis between the differential metabolites and clinical indictors. **J** ROC curves of the differential metabolites. **K** The relative abundance of metabolites with AUC > 0.7. ^*^P < 0.05, ^**^P < 0.01, ^***^P < 0.001

To elucidate the distinct metabolic processes implied by transcriptomics, we collected plasma from 30 PRs and 20 GRs for metabolomics, as illustrated in Fig. 3A. Figure 3B presents the PCA analysis. Subsequent annotation of these metabolites revealed a significant emphasis on lipid and lipid-related molecules (Fig. 3C). A total of 71 metabolites exhibited significantly altered levels between the PR and GR groups (VIP > 1 and p < 0.05, Fig. 3D), and their expression is depicted in Fig. 3E. Mapping these metabolites to canonical metabolic pathways revealed predominant alterations in pathways associated with bile acid biosynthesis, beta-oxidation of long-chain fatty acids, and fatty acid biosynthesis (Fig. 3F). Elevated bilirubin is a clinical feature of PBC patients. Under healthy conditions, the liver eliminates bilirubin through glucuronidation in hepatocytes, and its elevation indicates liver dysfunction. Bile acid biosynthesis is intricately linked to fatty acid metabolism through shared intermediates. Among all significantly altered metabolites, 39 were lipid and lipid-related molecules. The liver, a major organ for complex lipid biosynthesis, plays a central role in lipoprotein synthesis. Dysregulation of these lipid species suggests that lipid acid biosynthesis could impact drug response in these patients.

To identify metabolites serving as predictors of patient response, we conducted joint pathway analysis based on DE genes and metabolites. The results demonstrated impaired fatty acid degradation and biosynthesis in PR groups (Fig. 3G), with the metabolites involved in these processes listed in Fig. 3H. To assess the association between these metabolites and liver function, we performed correlation analysis with clinical indicators. The results showed that these lipid species were predominantly positively associated with impaired liver function or biliary tract issues (elevated ALP and GGT levels, Fig. 3I). AUC, sensitivity, and specificity were calculated to evaluate the discriminatory performance of each metabolite (Fig. 3J and Additional file 1: Fig. S2C). The relative abundance of metabolites with AUC > 0.7, including phosphatidylcholine (PC) (18:2/18:2), PC (16:0/20:3), PC (17:0/18:2), phosphatidylethanolamine (PE) (18:0e/20:4), PC (18:1e/22:6), sphingomyelin (SM) (d14:2/26:0), is shown in Fig. 3K. This group of metabolites is also included as potential predictors in the subsequent analysis.

### Involvement of Th cells and related cytokines in the response of VS patients

**Fig. 4:**
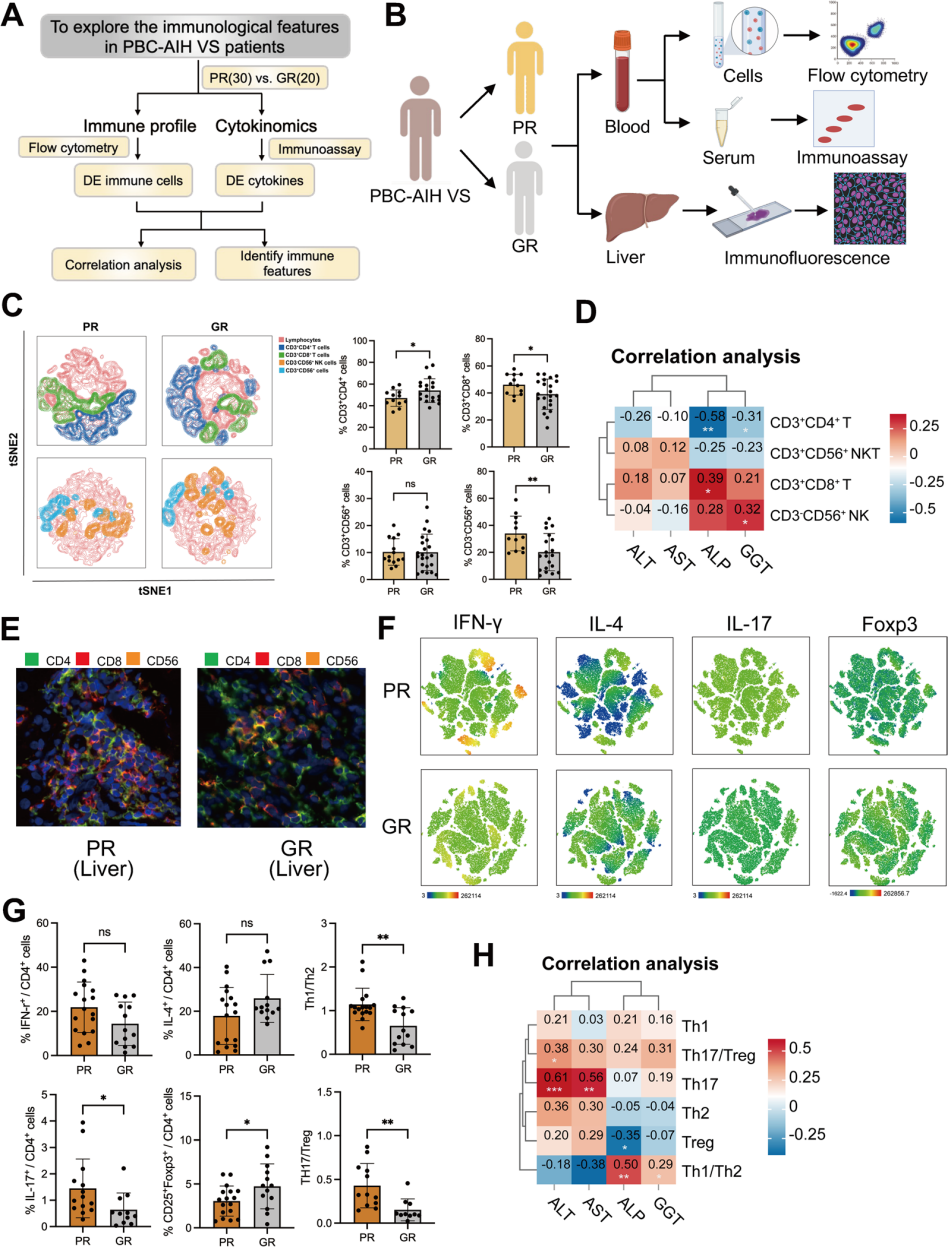
Immune cells alterations between the PR and GR groups. **A**, **B** The flow chart of the study design. **C** The proportion of CD3^+^CD4^+^ T cells, CD3^+^CD8^+^ T cells, CD3^−^CD56^+^ NK cells and CD3^+^CD56^+^ NKT cells in peripheral blood from PR and GR groups. **D** Correlation analysis between the immune cells above and clinical indictors. **E** Multiplex immunofluorescence (MIF) staining of liver tissues from GR and PR. Green represent CD4. Red represent CD8. Orange represent CD56. **F**, **G** The percentage of IFN-γ^+^ Th1 cells, IL4^+^ Th2 cells, IL17^+^ Th17 cells, Fxop3^+^ Tregs in between the PR and GR groups. **H** Correlation analysis between these cells and clinical parameters. ^*^P < 0.05, ^**^P < 0.01

Given the enriched presence of immune response and T-cell activities pathways in our transcriptomic data, we conducted a comprehensive exploration of immune cells and related cytokines that might play pivotal roles in disease progression (Fig. 4A). Employing flow cytometry and multi-immunofluorescence, we detected immune cell profiles in the liver and peripheral blood, while immunoassay technology evaluated 12 cytokines within the plasma (Fig. 4B). T cells and NK cells, the main representatives of adaptive and innate immune responses, were characterized by CD3 and CD56, respectively [54]. CD4 primarily represented Th cells, and CD8 represented Teff cells [55, 56]. Our results demonstrated a significant increase in the proportion of CD3^+^CD4^+^ Th cells and a significant decrease in CD3^+^CD8^+^ Teff cells and CD3^−^CD56^+^ NK cells in the peripheral blood and liver of the GR group compared to the PR group (Fig. 4C and E). CD3^+^CD56^+^ NKT cells showed no significant difference between the two groups. Correlation analysis revealed that the percentage of Th cells was negatively associated with liver function (ALP and GGT levels, Fig. 4D), suggesting the positive roles of these cells in drug response. To elucidate the specific subpopulations of Th cells involved in disease progression, we further analyzed different subpopulations using IFN-r for Th1 cells, IL-4 for Th2 cells, IL-17 for Th17 cells, and Foxp3 for regulatory T (Treg) cells [57]. Our results showed an upregulation of anti-inflammatory Th2 cells and Tregs in the GR group compared to the PR group, while pro-inflammatory Th1 and Th17 cells were downregulated in the GR group (Fig. 4F and G). Further analysis revealed significantly downregulated ratios of Th1 to Th2 and Th17 to Treg in the GR groups, suggesting that the dynamic balance between Th cells collectively influences disease progression and patient response (Fig. 4G). Correlation analysis showed a positive correlation between these pro-inflammatory Th cells and the severity of liver function, and a negative correlation between anti-inflammatory Th cells (Fig. 4H), further confirming that the dynamic balance of these cells influences patient liver function.

**Fig. 5:**
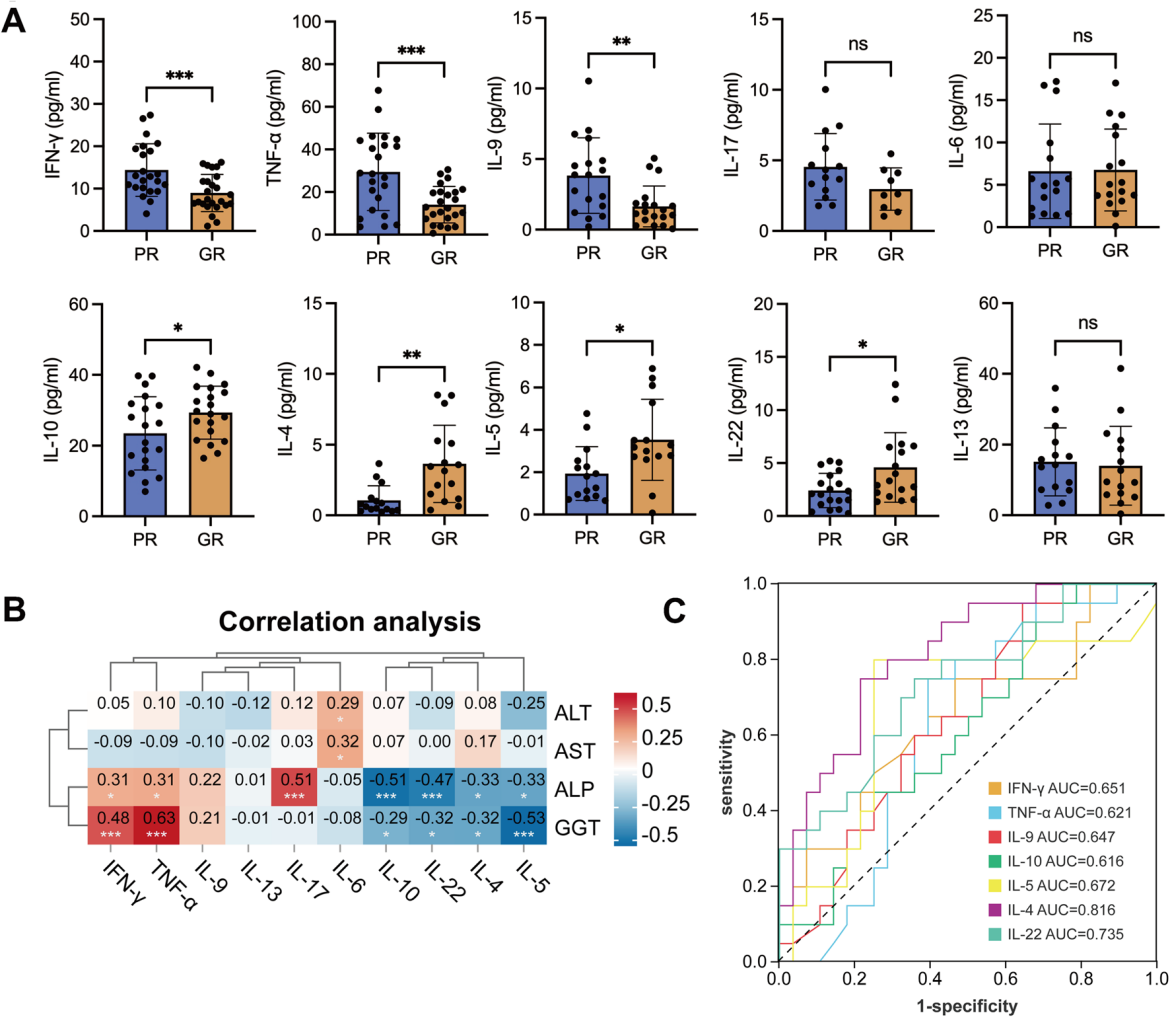
Cytokine profiling in plasma from patients with PBC/AIH VS. **A** Plasma levels of different cytokines between the PR and GR groups. **B** Correlation analysis between the levels of cytokines and clinical parameters. **C** ROC curves of the significant differential expressed cytokines. ^*^ P < 0.05, ^**^P < 0.01

Th cells produce cytokines in response to immune stimuli, mediating inflammation and modulating other immune cells. Understanding cytokine regulation and function has offered innovative treatment for many human diseases [58]. To this end, we evaluated Th-related cytokines within the plasma of our patients. The results showed that pro-inflammatory cytokine levels, including IFN-γ, TNF-α, IL-9, and IL-17, were downregulated in the GR group, while anti-inflammatory cytokine levels, including IL-10, IL-4, IL-5, and IL-22, were elevated in the GR group (Fig. 5A), suggesting the involvement of cytokines in the drug response of PBC/AIH patients. Correlation analyses demonstrated the negative association between anti-inflammatory cytokines and liver function indexes (Fig. 5B), indicating their protective roles in disease development. To ascertain whether these cytokines could serve as predictors of patient response, AUC was performed, with IL-4 and IL-22 showing good predictive ability (AUC > 0.7, Fig. 5C).

### Construction of ceRNA networks involved in metabolic and immune regulation

**Fig. 6:**
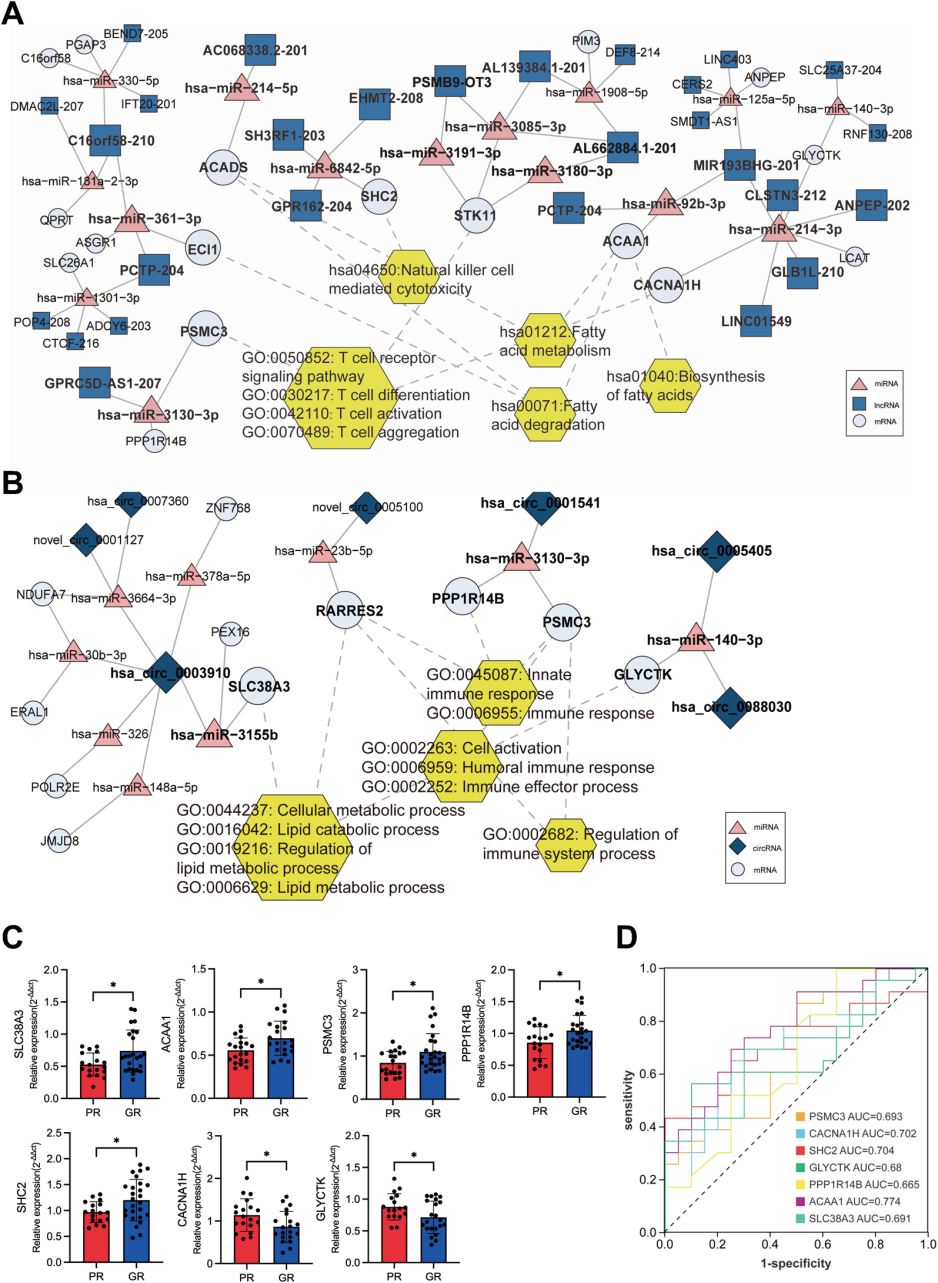
Construction of ceRNA networks related to metabolic and immune pathways. **A** LncRNA-related ceRNA networks. Pink triangles represent miRNAs. Light blue squares represent lncRNAs. White circles represent mRNAs, including PSMCS, ECl1, ACADS, SHC2, STK11, ACAA1 and CACNA1H. **B** CircRNA-related ceRNA networks. Pink triangles represent miRNAs. Dark blue prisms represent circRNAs. White circles represent mRNAs, including SLC38A3, RARRES2, PPP1R14B, PSMC3 and GLYCTK. **C** Expression of the target genes in the lncRNA/circRNA-related networks. **D** ROC curves of the significant differential expressed genes. ^*^P < 0.05

We integrated mRNA, miRNA, lncRNA, and circRNA data from whole-transcriptome sequencing to formulate ceRNA regulatory networks. This effort resulted in the construction of 256 lncRNA-miRNA-mRNA networks, encompassing 185 lncRNAs, 65 miRNAs, and 70 mRNAs, and 19 circRNA-miRNA-mRNA networks comprising 13 circRNAs, 11 miRNAs, and 15 mRNAs (Additional file 1: Fig. S3A and S3B). Analyzing these networks using GO and KEGG pathway analysis revealed significant enrichment of target genes within metabolic and immune response pathways. In the functional analysis of lncRNA-associated ceRNA networks, PSMC3, STK11, and CACNA1H were implicated in T-cell-related activities, SHC2 in NK-cell function, while ECl1, ACADS, and ACAA1 were linked to fatty acid metabolism (Fig. 6A). For circRNA-associated ceRNA networks, SLC38A3, RARRSE2, and GLYCTK were involved in lipid metabolic processes, and PSMC3, PPP1R14B, and RARRSE2 were associated with immune response pathways (Fig. 6B). The gene patterns from our transcriptomic data are illustrated in Additional file 1: Fig. S4A. To elucidate the regulatory molecules for these genes, we identified genes with expression trends opposite to their corresponding miRNAs and scrutinized the co-expression patterns of mRNA-lncRNA and mRNA-circRNA pairs. Significant networks were illustrated in Fig. 6A and B (COR > 0.85 and p < 0.05). Genes encode proteins, forming a fundamental link between genomic information and cellular processes. We expanded the sample size to verify whether these genes exhibited differences in the PR and GR groups. Results revealed significant upregulation in the GR group compared to the PR group for SLC38A3, ACAA1, PSMC3, PPP1R14B, and SHC2, while CACNA1H and GLYCTK were downregulated (Fig. 6C), suggesting the involvement of these genes in patient response. The remaining genes showed no significance, likely attributed to the limited sample size (Additional file 1: Fig. S4B). AUC analysis demonstrated that CACNA1H, SHC2 and ACAA1 had good predictive ability (AUC > 0.7, Fig. 6D). Due to the limited sample size, further confirmation of the ceRNA network's modulation of patient response was not feasible, but these findings provide potential targets for subsequent mechanistic studies.

### Prediction of biochemical response in PBC/AIH VS

**Fig. 7:**
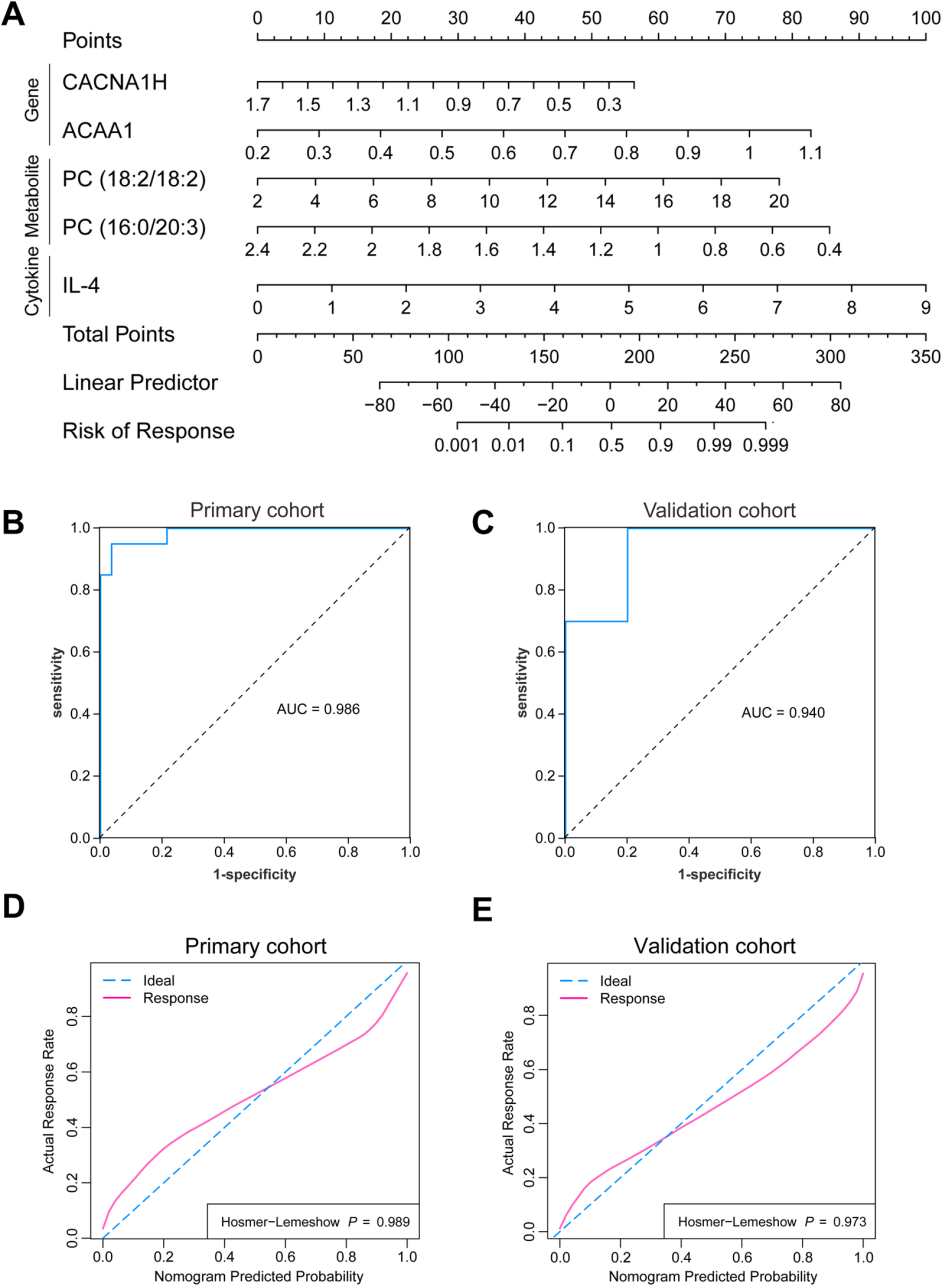
Performance of the combined model for prediction of biochemical response. **A** Nomogram based on the combined model to predict biochemical response in PBC/AIH VS patients. **B**, **C** ROC curves showing the performance of the combined model in primary cohort and validation cohort. **D**, **E** The calibration curve of the combined model in primary cohort and validation cohort

To identify reliable biomarkers for predicting biochemical response in PBC/AIH VS patients, we conducted a comprehensive analysis integrating data on differentially expressed metabolites, cytokines, and genes. Significant predictors of complete treatment response in univariate analysis included ALP level, PC (18:2/18:2), PC (16:0/20:3), PC (17:0/18:2), PC (18:1e/22:6), SM (d14:2/26:0), IL-5, IL-4, IL-22, PSMC3, CACNA1H, SHC2, GLYCTK, PPP1R14B, ACAA1, and SLC38A3. However, in multivariate analysis, only PC (18:2/18:2) (p = 0.041), PC (16:0/20:3) (p = 0.043), IL-4 (p = 0.028), CACNA1H (p = 0.047), and ACAA1 (p = 0.046) emerged as significant predictors of complete response to treatment (Additional file 1: Table S5). A nomogram was constructed based on these indicators (Fig. 7A). ROC curves were employed to compare the predictive efficiencies of the combined model in the primary and validation cohorts. The combined model exhibited excellent prediction efficiency, with an AUC of 0.986 in the primary cohort and an AUC of 0.940 in the validation cohort for complete biochemical response prediction (Fig. 7B and C). The calibration curve, representing a visual nomogram, demonstrated near-perfect alignment, indicating robust agreement between prediction and observation (Fig. 7D and 7E). The H–L test confirmed the model's goodness of fit (p = 0.989 in the primary cohort; p = 0.973 in the validation cohort).

## Discussion

PBC/AIH VS, a morbid condition within autoimmune liver diseases, poses challenges for rigorous study due to its rarity in the general population. Despite numerous small-scale studies over recent decades, uncertainty persists regarding optimal treatment strategies for this syndrome. While UDCA and corticosteroids, with or without AZA, have been acknowledged as the first-line treatment for PBC/AIH VS, 40%-60% of patients exhibit inadequate biochemical responses, remaining at risk of progressing to advanced disease stages, such as liver fibrosis and cirrhosis [20, 21]. Second-line therapies, including tacrolimus [13], mycophenolate mofetil (MMF) [59, 60], or cyclosporine [13, 15], are anticipated to enhance the prognosis and survival of patients within this subgroup. However, evaluating the biochemical response necessitates a wait of 6 months or more after treatment. Thus, there is an urgent need for predictive indicators to identify patients more likely to exhibit insufficient biochemical responses, facilitating early access to additional treatment. In this preliminary study, we observed significant alterations in transcript levels associated with metabolic and immune responses in the livers of patients in the PR and GR groups via whole transcriptome analysis. Furthermore, we elucidated specific metabolic pathways, metabolites, and immune cytokines through metabolomic and cytokinomic analyses. Lipid species (PC (18:2/18:2) and PC (16:0/20:3)), cytokines (IL-4), and genes (CACNA1H and ACAA1) were identified as indicative of insufficient biochemical responses in PBC/AIH VS patients. The combined model, incorporating these five indicators, demonstrated proficiency in predicting the risk of insufficient response in PBC/AIH VS.

In our transcriptome analysis, the target genes CACNA1H and ACAA1 exhibited significant expression differences between the PR and GR groups. Functional analysis revealed that CACNA1H was primarily associated with T cell differentiation, activation, and aggregation, while ACAA1 was enriched in fatty acid metabolism. CACNA1H encodes a protein for the α1 subunit of voltage-gated calcium channels, playing a regulatory role in calcium ion entry into cells. Its presence spans all CD4^+^ T cell subsets, including Th1, Th2, Th17, and Tregs [61], modulating T cell expansion and apoptosis through voltage-gated calcium channel function. Notably, CACNA1C and CACNA1G, encoding α1 channel subunits, have been linked to Th2 and Th17 cell function, respectively, through voltage-activated calcium influx [62, 63]. AIH and PBC are characterized by T cell-mediated autoimmune responses against liver autoantigens with distinct patterns of destruction [1, 2]. Hence, CACNA1H may contribute to the patient response process by modulating T cell activity, although more in-depth studies are warranted. ACAA1, or acetyl-CoA acyltransferase 1, serves a key role in lipid metabolism, specifically in the beta-oxidation of fatty acids within mitochondria [64]. Dysregulation of ACAA1 is associated with disturbances in lipid metabolism, implicating its role in various metabolic disorders [65]. Altered ACAA1 expression leads to hepatic lipid metabolism abnormalities in our PBC/AIH VS patients, influencing their drug response. The ceRNA network constitutes a sophisticated regulatory network encompassing diverse RNA molecules vying for binding to shared miRNAs. Within this intricate interplay, lncRNAs, circRNAs, and mRNAs harbor MREs, enabling them to sequester miRNAs and consequently exert influence on each other's expression levels [25]. This competitive binding gives rise to a network wherein changes in one RNA type can impact the abundance and function of others within the network [66]. The ceRNA hypothesis posits that these molecules engage in crosstalk, thereby contributing to the regulation of gene expression and cellular processes. Our findings showcase the involvement of a ceRNA network in the regulation of target genes, encompassing lncRNAs, circRNAs, and miRNAs. However, owing to the limitations of the sample size, further validation of these regulatory mechanisms was unattainable in this study, underscoring the need for future investigations in this direction.

The liver plays a central role in lipid metabolism, encompassing functions ranging from lipid synthesis and storage to lipid breakdown and energy release. Our metabolomic data unveil dysregulated lipid metabolism between the PR and GR groups, suggesting its implication in the response of our patients. Levels of ALP and GGT in our patients were significantly higher in the PR group than in the GR group. ALP and GGT, enzymes primarily localized in the liver, are involved in bile acid metabolism. Dysregulation of bile acid metabolism affects the digestion and absorption of lipids in the intestinal tract, thereby contributing to the differences in lipid molecules between the two groups. These lipid molecules have been reported to participate in the progression of various diseases and are regarded as biomarkers [67, 68]. Our results identified PC (18:2/18:2) and PC (16:0/20:3) as suggestive predictors for predicting the risk of insufficient response in PBC/AIH VS, indicating their involvement in disease development. Additionally, lipid species have been reported to participate in various cellular activities, providing energy and serving as signaling molecules. Our previous study found that lipid species, including lysophosphatidylcholine (LPC) 16:0, 18:1, 18:2, and 18:3, were associated with monocyte activation in AIH [69]. However, whether PC (18:2/18:2) and PC (16:0/20:3) can regulate the immune response to participate in disease development requires further investigation.

PBC/AIH VS is characterized by a T cell–mediated autoimmune response against liver autoantigens, yet its immunological foundations are largely unexplored. Our findings revealed a significant Th1/Th2 and Th17/Treg cell immune imbalance in both PR and GR groups. Th1 and Th17 cells, secreting IFN-γ and IL-17, contribute to the inflammatory response, while Th2 cells, secreting IL-4, and Tregs play a critical role in maintaining immune tolerance and preventing autoimmunity by suppressing excessive immune responses. The balance and coordination of these Th cell subsets are crucial for a well-regulated immune system, and their dysregulation can lead to various immunological disorders and diseases [68]. The majority of VS patients exhibited AIH-like signatures, characterized by a more prominent inflammatory cytokine signature with the highest levels of IFN-γ, TNF-α, IL-9, and IL-17, aligning with AIH data from others [70, 71]. Additionally, our results identified IL-4 as a significant predictor of biochemical response to treatment in multivariate analysis. IL-4, a multifunctional cytokine produced by various immune cells, exerts its effects by binding to the IL-4 receptor and prompting the differentiation of naive T cells into Th2 cells. IL-4 possesses anti-inflammatory properties by inhibiting the production of pro-inflammatory cytokines and promoting the activity of Tregs, crucial for maintaining immune homeostasis [72]. Elevated levels of IL-4 and Th2 cells collectively suppress inflammation in patients, potentially facilitating biochemical remission, though further investigation is warranted.

We developed a nomogram to visually represent and validate the predictive capability of the combined model based on the aforementioned indicators. Despite the study's relatively modest sample size, the findings are promising. The multi-omics-based combined model demonstrated excellent predictive efficacy, exhibiting an AUC of 0.986 in the primary cohort and 0.940 in the validation cohort for anticipating complete biochemical response. This suggests that genes, metabolites, and cytokines may offer insights into pathological changes associated with the disease process earlier than conventional laboratory markers. Previous investigations predominantly concentrated on assessing biochemical response in PBC/AIH VS patients through clinical symptoms, pathological staging, and biochemical indicators [12]. To our knowledge, our study represents the first attempt to utilize multi-omic features for predicting insufficient biochemical response in PBC/AIH VS patients. Our findings hold significant generalizability and application potential. The selection of pretreatment indicators can aid in identifying high-risk patients with a poor response, enabling the implementation of early interventions.

This study is subject to several limitations. Firstly, the retrospective design introduces significant selection bias, potentially hindering the accurate representation of real clinical conditions. Secondly, the limited sample size and challenges in sampling led to most patients being assessed for metabolism and cytokines only prior to treatment, with a lack of gene expression testing. Future investigations should prospectively involve a larger sample size to identify more robust indicators for predicting biochemical responses in patients with PBC/AIH VS. Subsequent large-scale multicenter studies are essential to validate our model. Lastly, the absence of suitable animal models prevented further exploration of the identified indicators' role in disease development. Further validation of the underlying mechanisms is warranted in future studies.

In conclusion, our integrated analysis of whole transcriptomics, metabolomics, and cytokineomics revealed substantial alterations in lipid metabolism and immune responses, particularly in Th cells and their associated factors among patients with PBC/AIH VS. We identified ACAA1 and CACAN1H genes that likely play regulatory roles in these processes. The amalgamation of these features allowed us to construct a predictive model, suggesting an insufficient biochemical response in PBC/AIH patients post-treatment. Moreover, a nomogram incorporating potential risk factors emerges as a valuable tool for clinicians, enabling the early identification of patients with insufficient response and facilitating timely interventions.

### Supplementary Information

**Additional file 1: Table S1.** Demographic and clinical features of the primary cohort. **Table S2.** Comparison of baseline clinical features of poor and good responders in primary cohort. **Table S3.** Comparison of baseline clinical features of primary cohort and validation cohort. **Table S4.** Primer sequences of genes. **Table S5.** Univariate and multivariate logistic regression analysis results. **Figure S1.** A histological features of a PBC-AIH OS patient: Prominent interface hepatitis with numerous plasma cells and typical rosetting of hepatocytes. **Figure S2.** (A) Correlation coefficient between samples. (B) PCA analysis between the PR and GR groups. (C) ROC curves of the differential metabolites. **Figure S3.** (A) Construction of lncRNA-related ceRNA networks. Pink triangles represent miRNAs. Light blue squares represent lncRNAs. White circles represent mRNAs. (B) Construction of circRNA-related ceRNA networks. Pink triangles represent miRNAs. Dark blue prisms represent circRNAs. White circles represent mRNAs. **Figure S4.** (A)The genes expression in our transcriptomic data. (B) The validation of rest of involved genes with no significance.

## Data Availability

The data presented in this current study are available from the corresponding authors on reasonable request.
