# Supplementary material for: Multi-omics approaches for drug-response characterization in primary biliary cholangitis and autoimmune hepatitis variant syndrome
Source: J Transl Med. 2024 Feb 29;22:214. doi: 10.1186/s12967-024-05029-6 (PMC10902991; doi:10.1186/s12967-024-05029-6)

**Table S1: Demographic and clinical features of the primary cohort.**

| Parameters | PBC/AIH VS (n=50) |
| --- | --- |
| Age | 49.08±10.63 |
| Female, n (%) | 43 (86.0) |
| ALT | 160.52±94.18 |
| AST | 180.12±120.64 |
| ALB | 38.64±6.34 |
| GLB | 39.85±7.51 |
| ALP | 391.10±204.71 |
| GGT | 485.83±369.75 |
| IGG | 26.78±27.71 |
| IGM | 3624.66±2496.32 |
| TBIL | 59.38±64.05 |
| ANA positive, n (%) | 46 (92.0) |
| AMA positive, n (%) | 25(50.0) |

PBC: Primary biliary cholangitis; AIH: Autoimmune hepatitis; VS: Variant syndrome; ALT: Alanine aminotransferase; AST: Aspartate aminotransferase; ALB: albumin; GLB: Globulin; ALP: Alkaline phosphatase; GGT: g-glutamyl transpeptidase; IgG: Immunoglobulin G; IgM: Immunoglobulin M; TBIL: Total bilirubin; AMA: Anti-mitochondrial antibody; ANA: Antinuclear antibody.

**Table S2: Comparison of baseline clinical features of poor and good responders in primary cohort.**

| Parameters | Poor responder (n=30) | Good responder (n=20) | P value |
| --- | --- | --- | --- |
| Age | 46.23±9.54 | 53.35±10.99 | 0.019 |
| Female, n (%) | 24 (80.0) | 19 (95.0) | 0.279 |
| ALT | 151.27±77.25 | 174.40±115.88 | 0.400 |
| AST | 154.14±71.75 | 219.10±164.34 | 0.061 |
| ALB | 38.55±6.26 | 38.78±6.63 | 0.903 |
| GLB | 38.81±7.63 | 41.43±7.23 | 0.231 |
| ALP | 441.83±229.64 | 315.00±131.97 | 0.030^*^ |
| GGT | 563.63±421.49 | 369.10±239.82 | 0.044^*^ |
| IGG | 27.84±35.62 | 25.19±6.32 | 0.055 |
| IGM | 3742.10±2594.47 | 3448.5±2396.36 | 0.527 |
| TBIL | 66.95±65.83 | 48.03±61.16 | 0.311 |
| ANA positive, n (%) | 28 (93.3) | 18 (90.0) | 0.670 |
| AMA positive, n (%) | 16 (53.3) | 9 (45.0) | 0.564 |

PBC: Primary biliary cholangitis; AIH: Autoimmune hepatitis; VS: Variant syndrome; ALT: Alanine aminotransferase; AST: Aspartate aminotransferase; ALB: albumin; GLB: Globulin; ALP: Alkaline phosphatase; GGT: g-glutamyl transpeptidase; IgG: Immunoglobulin G; IgM: Immunoglobulin M; TBIL: Total bilirubin; AMA: Anti-mitochondrial antibody; ANA: Antinuclear antibody.

**Table S3: Comparison of baseline clinical features of primary cohort and validation cohort.**

| Parameters | Primary cohort (n=50) | Validation cohort (n=20) | P value |
| --- | --- | --- | --- |
| Age | 49.08±10.63 | 45.8±11.78 | 0.262 |
| Female, n (%) | 43 (86.0) | 18 (90.0) | 0.955 |
| ALT | 160.52±94.18 | 160.36±86.33 | 0.995 |
| AST | 180.12±120.64 | 162.96±102.50 | 0.577 |
| ALB | 38.64±6.34 | 41.16±5.66 | 0.127 |
| GLB | 39.85±7.51 | 42.24±7.28 | 0.231 |
| ALP | 391.10±204.71 | 421.75±239.95 | 0.592 |
| GGT | 485.83±369.75 | 581.88±416.31 | 0.347 |
| IGG | 26.78±27.71 | 23.58±6.75 | 0.613 |
| IGM | 3624.66±2496.32 | 3942.00±3275.56 | 0.663 |
| TBIL | 59.38±64.05 | 34.39±20.62 | 0.093 |
| ANA positive, n (%) | 46 (92.0) | 18 (90.0) | 0.458 |
| AMA positive, n (%) | 25(50.0) | 8 (40.0) | 0.623 |

PBC: Primary biliary cholangitis; AIH: Autoimmune hepatitis; VS: Variant syndrome; ALT: Alanine aminotransferase; AST: Aspartate aminotransferase; ALB: albumin; GLB: Globulin; ALP: Alkaline phosphatase; GGT: g-glutamyl transpeptidase; IgG: Immunoglobulin G; IgM: Immunoglobulin M; TBIL: Total bilirubin; AMA: Anti-mitochondrial antibody; ANA: Antinuclear antibody.

**Table S4: Primer sequences of genes.**

| Gene Symbol | Item | Sequence (5′ -> 3′) |
| --- | --- | --- |
| ACADS | Forward Primer | CGGCAGTTACACACCATCTAC |
|  | Reverse Primer | GCAATGGGAAACAACTCCTTCTC |
| ECl1 | Forward Primer | TGACCTGTGACTACCGCATC |
|  | Reverse Primer | ATGGTGTTCTCCAGGGTGTC |
| STK11 | Forward Primer | CTGAGTACGAACCGGCCAA |
|  | Reverse Primer | CTACGGCACCACAGTCATG |
| SLC38A3 | Forward Primer | ATCTCCAACCTGTCCATCGC |
|  | Reverse Primer | GCCAACGGCAATAAGCACAT |
| PPP1R14B | Forward Primer | CCCACAGAGGCCTTCATTT |
|  | Reverse Primer | TGCTGTTGCTACGAGGTTG |
| GLYCTK | Forward Primer | TGGGCCAGCATCTTGTG |
|  | Reverse Primer | GAATACCTGGACACGGCTATG |
| RARRES2 | Forward Primer | CTCTGAGGACAAAGTTCTGGG |
|  | Reverse Primer | CAGGGAAGTAGAAGCTGTGG |
| PSMC3 | Forward Primer | TGGTGCAGATGTTCATTGGAG |
|  | Reverse Primer | CCAGCCTTCTCACTGTCAAAG |
| SHC2 | Forward Primer | TGGACTTTAACACGCGCA |
|  | Reverse Primer | AAGGTTGCTCTTGCCCAG |
| ACAA1 | Forward Primer | CGGTTCTCAAGGACGTGAATC |
|  | Reverse Primer | TGACAGTGGACAAAGGCAC |
| CACNA1H | Forward Primer | TGACCTTCGGCAACTATGTG |
|  | Reverse Primer | GGAGTTCTCTGAGCTTGTGG |

**Table S5: Univariate and multivariate logistic regression analysis results**

| Predictors | ROC | Univariate logistic regression | | | Multivariate logistic regression | | |
| --- | --- | --- | --- | --- | --- | --- | --- |
|  | AUC | OR | 95% CI | P value | OR | 95% CI | P value |
| Clinical parameters | | | | | | | |
| ALT level > 40 (IU/L) |  | 1.097 | 0.859-1.399 | 0.459 |  |  |  |
| AST level > 35 (IU/L) |  | 1.190 | 0.978-1.449 | 0.083 |  |  |  |
| ALP level > 135 (IU/L) |  | 0.591 | 0.356-0.980 | 0.041 |  |  |  |
| GGT level > 45 (IU/L) |  | 0.927 | 0.849-1.012 | 0.089 |  |  |  |
| Metabolites | | | | | | | |
| PC (18:2/18:2) | 0.764 | 1.265 | 1.067-1.501 | 0.007 | 2.044 | 1.029-4.058 | 0.041 |
| PC (16:0/20:3) | 0.855 | 0.032 | 0.005-0.226 | 0.001 | 0.007 | 0.000-0.856 | 0.043 |
| PC (17:0/18:2) | 0.746 | 0.297 | 0.101-0.871 | 0.027 |  |  |  |
| PE (18:0e/20:4) | 0.746 | 0.743 | 0.544-1.014 | 0.061 |  |  |  |
| PC (18:1e/22:6) | 0.734 | 0.159 | 0.030-0.848 | 0.031 |  |  |  |
| SM (d14:2/26:0) | 0.743 | 1.404 | 1.077-1.830 | 0.012 |  |  |  |
| Cytokines | | | | | | | |
| IFN-γ | 0.651 | 0.903 | 0.808-1.011 | 0.076 |  |  |  |
| TNF-α | 0.621 | 0.962 | 0.922-1.004 | 0.075 |  |  |  |
| IL-9 | 0.647 | 0.744 | 0.548-1.008 | 0.056 |  |  |  |
| IL-10 | 0.616 | 1.056 | 0.984-1.133 | 0.130 |  |  |  |
| IL-5 | 0.672 | 1.387 | 1.003-1.917 | 0.048 |  |  |  |
| IL-4 | 0.816 | 1.960 | 1.261-3.048 | 0.003 | 4.144 | 1.171-14.669 | 0.028 |
| IL-22 | 0.735 | 1.655 | 1.129-2.426 | 0.010 |  |  |  |
| Gene expression | | | | | | | |
| PSMC3 | 0.693 | 43.355 | 2.271-827.530 | 0.002 |  |  |  |
| CACNA1H | 0.702 | 0.103 | 0.015-0.723 | 0.022 | 0.008 | 0.000-0.935 | 0.047 |
| SHC2 | 0.704 | 22.040 | 1.980-245.370 | 0.012 |  |  |  |
| GLYCTK | 0.68 | 0.015 | 0.001-0.282 | 0.005 |  |  |  |
| PPP1R14B | 0.668 | 54.256 | 2.401-1225.841 | 0.012 |  |  |  |
| ACAA1 | 0.774 | 9.871 | 2.152-45.283 | 0.003 | 15854.5 | 1.011-418094794 | 0.046 |
| SLC38A3 | 0.691 | 73.178 | 3.272-1636.723 | 0.007 |  |  |  |

**Figure S1:** A histological features of a PBC-AIH OS patient: Prominent interface hepatitis with numerous plasma cells and typical rosetting of hepatocytes.


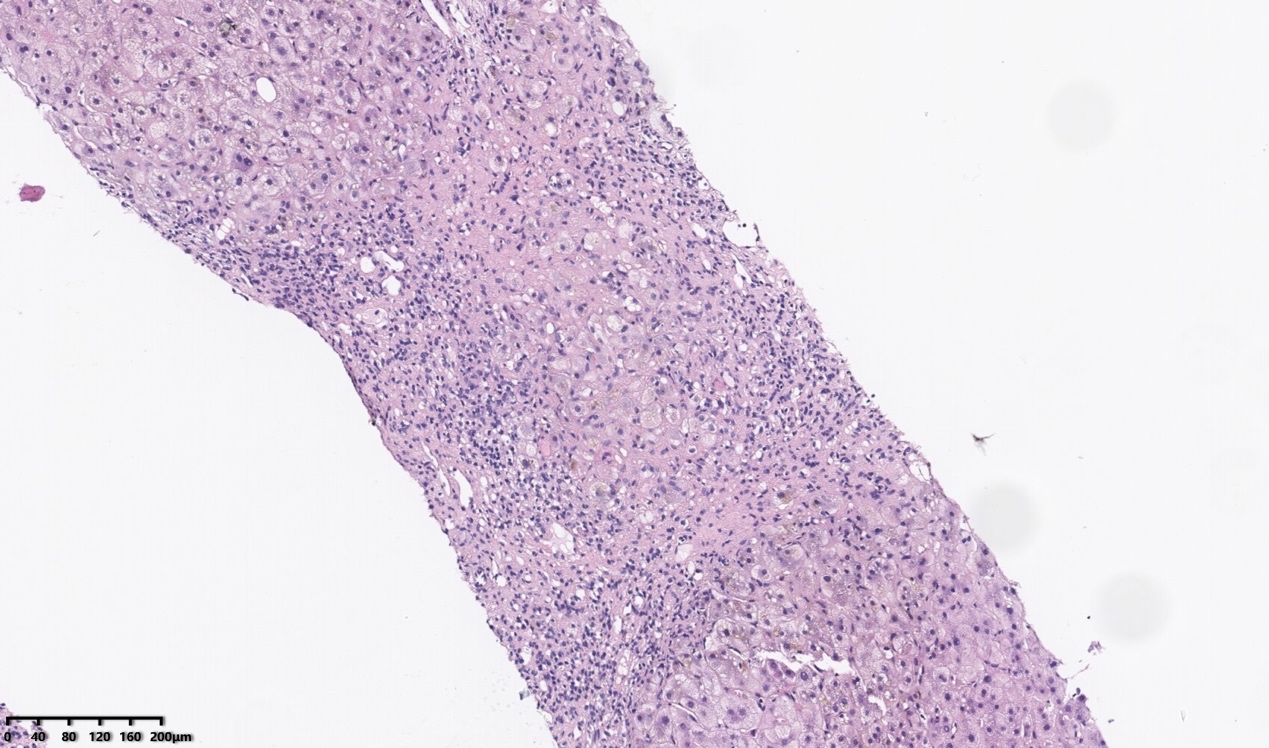


**Figure S2:** (A) Correlation coefficient between samples. (B) PCA analysis between the PR and GR groups. (C) ROC curves of the differential metabolites.


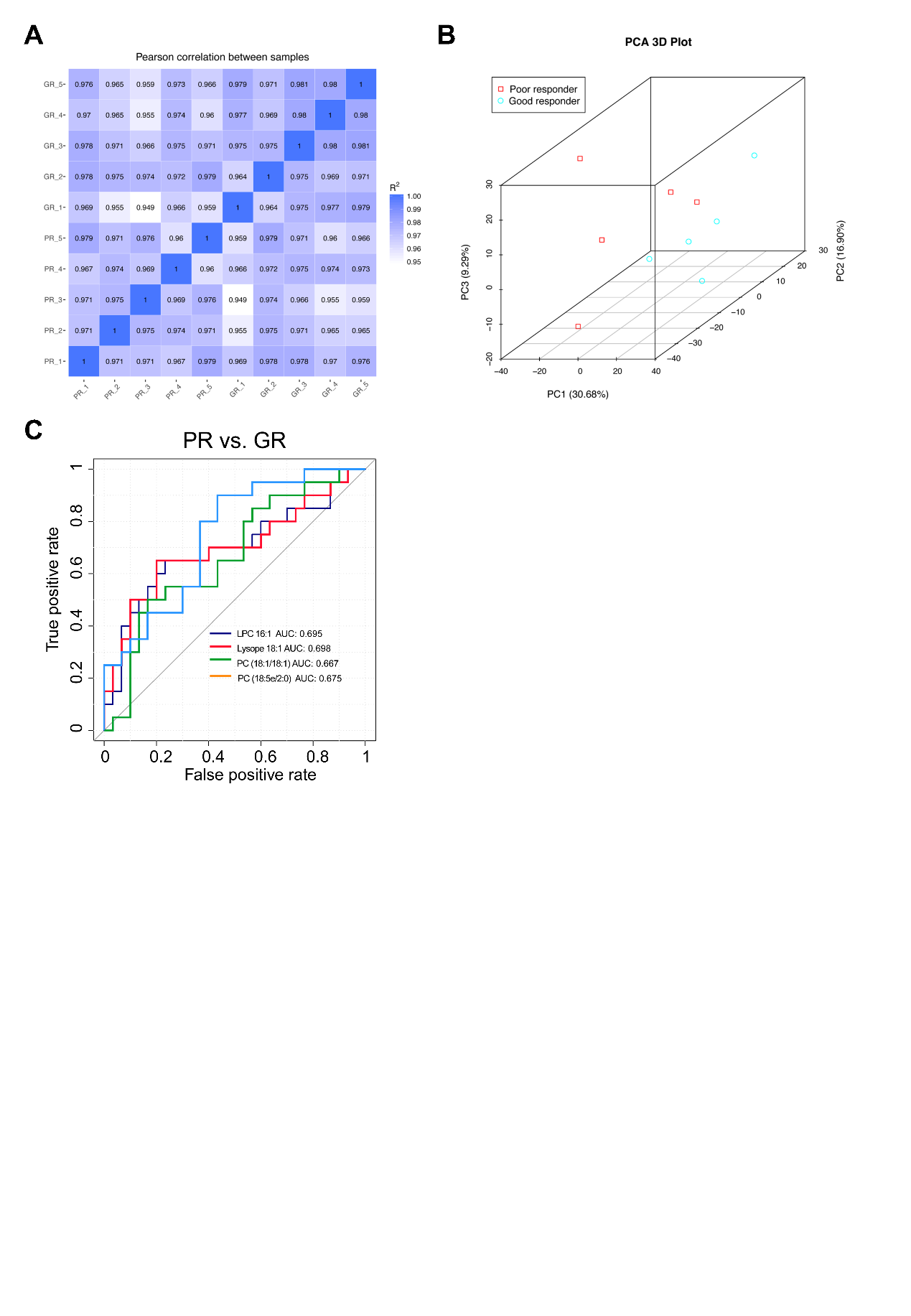


**Figure S3:** (A) Construction of lncRNA-related ceRNA networks. Pink triangles represent miRNAs. Light blue squares represent lncRNAs. White circles represent mRNAs. (B) Construction of circRNA-related ceRNA networks. Pink triangles represent miRNAs. Dark blue prisms represent circRNAs. White circles represent mRNAs.


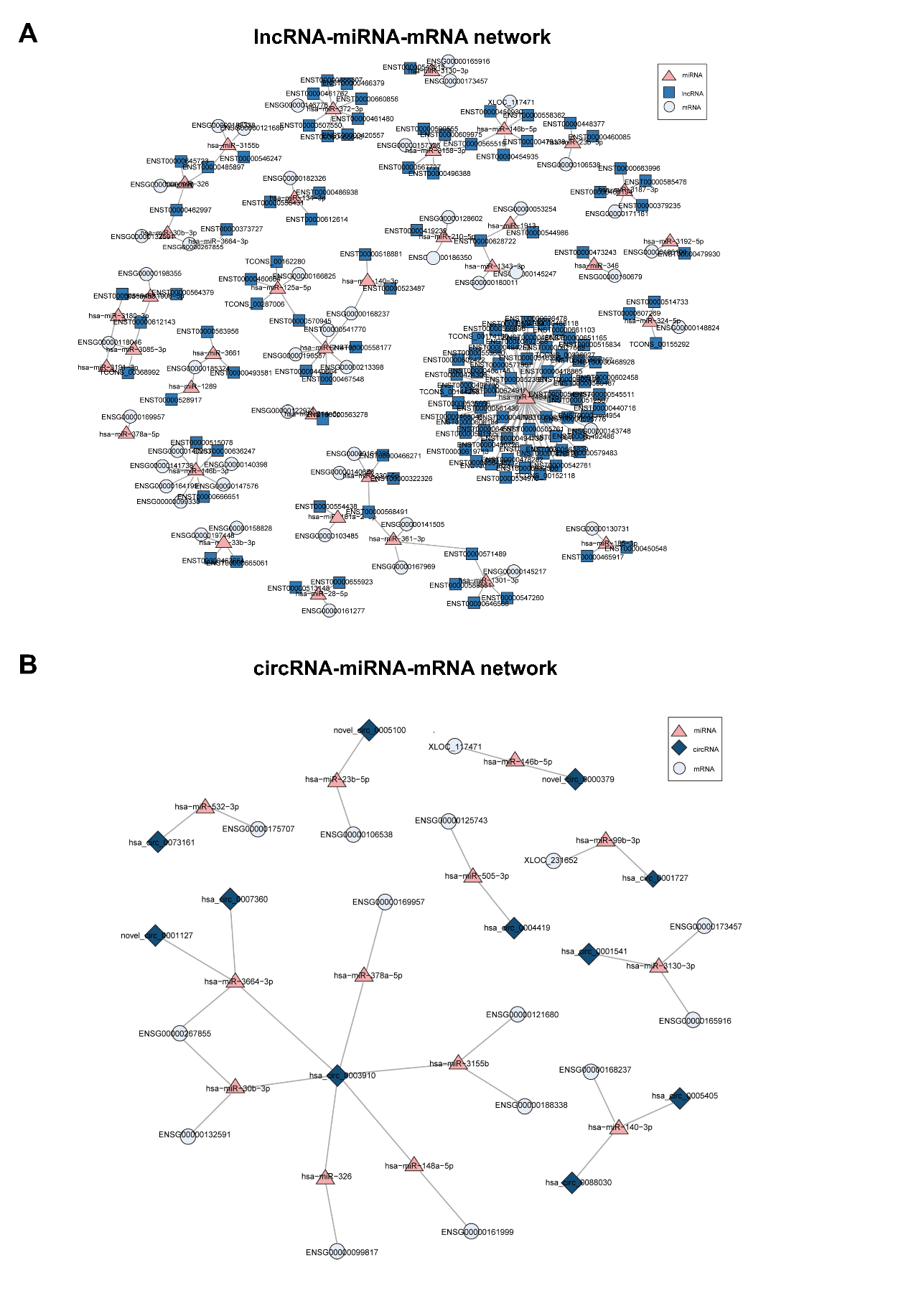


**Figure S4:** (A)The genes expression in our transcriptomic data. (B) The validation of rest of involved genes with no significance.


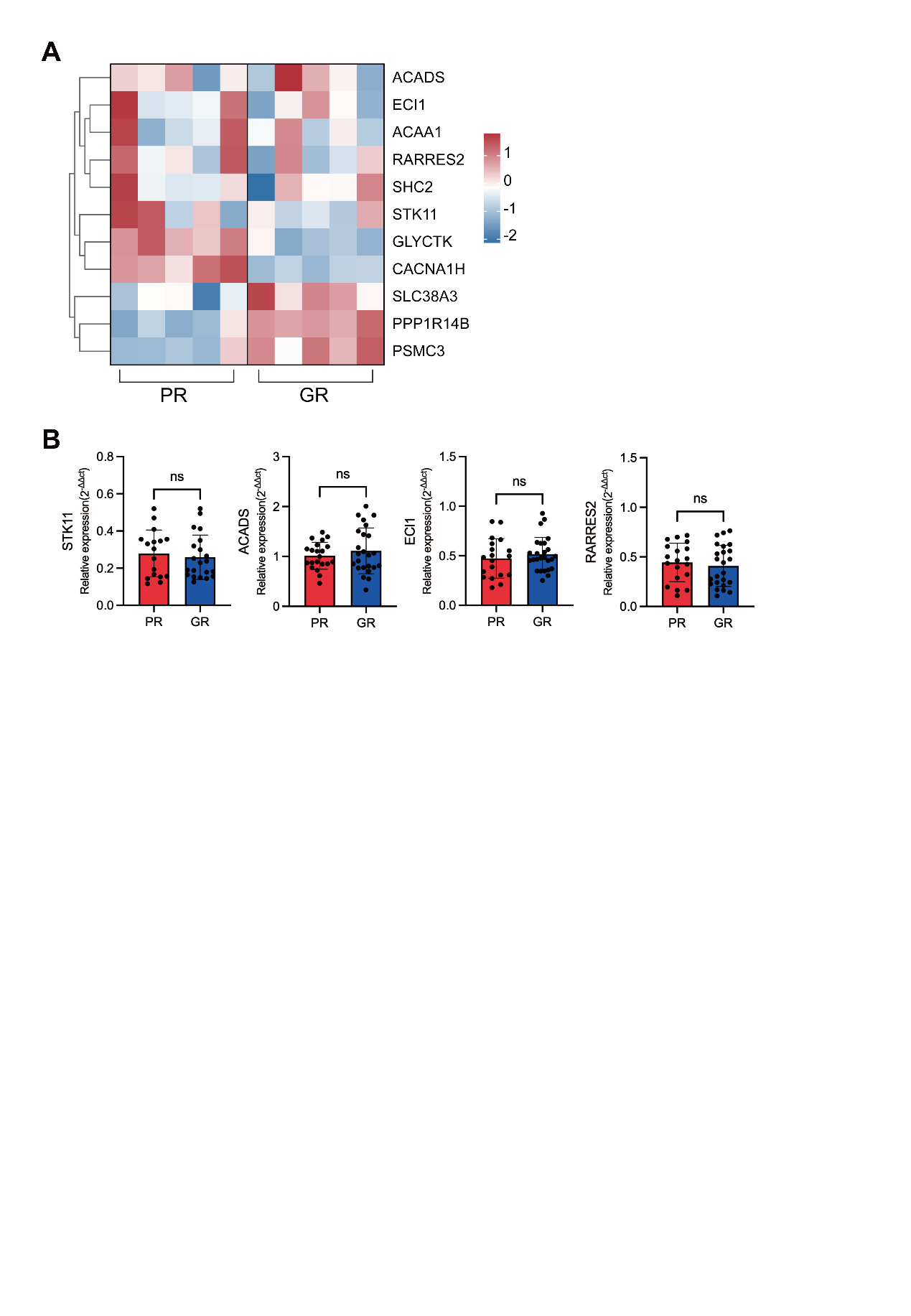

Supplement: Supplementary file 1 — Additional file 1: Table S1. Demographic and clinical features of the primary cohort. Table S2. Comparison of baseline clinical features of poor and good responders in primary cohort. Table S3. Comparison of baseline clinical features of primary cohort and validation cohort. Table S4. Primer sequences of genes. Table S5. Univariate and multivariate logistic regression analysis results. Figure S1. A histological features of a PBC-AIH OS patient: Prominent interface hepatitis with numerous plasma cells and typical rosetting of hepatocytes. Figure S2. (A) Correlation coefficient between samples. (B) PCA analysis between the PR and GR groups. (C) ROC curves of the differential metabolites. Figure S3. (A) Construction of lncRNA-related ceRNA networks. Pink triangles represent miRNAs. Light blue squares represent lncRNAs. White circles represent mRNAs. (B) Construction of circRNA-related ceRNA networks. Pink triangles represent miRNAs. Dark blue prisms represent circRNAs. White circles represent mRNAs. Figure S4. (A)The genes expression in our transcriptomic data. (B) The validation of rest of involved genes with no significance. [file 12967_2024_5029_MOESM1_ESM.docx]
